# Whole-slide image analysis outperforms micrograph acquisition for adipocyte size quantification

**DOI:** 10.1080/21623945.2020.1823139

**Published:** 2020-09-20

**Authors:** Anne S Maguire, Lauren N Woodie, Robert L Judd, Douglas R Martin, Michael W Greene, Emily C Graff

**Affiliations:** a Scott-Ritchey Research Center, College of Veterinary Medicine, Auburn University, Auburn, AL, USA; b Department of Anatomy, Physiology & Pharmacology, College of Veterinary Medicine, Auburn University, Auburn, AL, USA; c Department of Nutrition, Dietetics, and Hospitality Management, College of Human Sciences, Auburn University, Auburn, AL, USA; d Institute for Diabetes, Obesity and Metabolism, Division of Endocrinology, Diabetes and Metabolism, Department of Medicine, University of Pennsylvania Perelman School of Medicine, Philadelphia, PA, USA; e Department of Pathobiology, College of Veterinary Medicine, Auburn University, Auburn, AL, USA

**Keywords:** Adipose tissue, obesity, diabetes, hypertrophy, hyperplasia

## Abstract

The distinction between biological processes of adipose tissue expansion is crucial to understanding metabolic derangements, but a robust method for quantifying adipocyte size has yet to be standardized. Here, we compared three methods for histological analysis *in situ*: one conventional approach using individual micrographs acquired by digital camera, and two with whole-slide image analysis pipelines involving proprietary (Visiopharm) and open-source software (QuPath with a novel ImageJ plugin). We found that micrograph analysis identified 10–40 times fewer adipocytes than whole-slide methods, and this small sample size resulted in high variances that could lead to statistical errors. The agreement of the micrograph method to measure adipocyte area with each of the two whole-slide methods was substantially less (R^2^ of 0.6644 and 0.7125) than between the two whole-slide methods (R^2^ of 0.9402). These inconsistencies were more pronounced in samples from high-fat diet fed mice. While the use of proprietary software resulted in the highest adipocyte count, the lower cost, ease of use, and minimal variances of the open-source software provided a distinct advantage for measuring the number and size of adipocytes. In conclusion, we recommend whole-slide image analysis methods to consistently measure adipocyte area and avoid unintentional errors due to small sample sizes.

## 1. Introduction

Adipose tissue is an extensive and versatile organ; it supports structures throughout the body and has a profound capacity to expand and shrink in response to metabolic demand. Physiologically, adipose tissue responds to excess energy by increasing cell size (hypertrophy), increasing cell number (hyperplasia), or a combination of both processes [1]. Metabolic derangements such as over-nutrition, insulin resistance, dyslipidemia, and altered immune function can result in an abnormal ratio of hypertrophic and hyperplastic adipocytes, which in turn negatively affects the function of adipose tissue mass [2,3]. Discussion in the literature has centred around whether hypertrophy or hyperplasia is most responsible for insulin resistance and type 2 diabetes mellitus [4–7], and recent reviews discuss the benefits of adipocyte hyperplasia [8] as well as unique mechanisms associated with adipogenesis [9]. Despite the interest in and importance of adipocyte hypertrophy and hyperplasia in homoeostasis and disease pathology, thorough investigations have been limited by the lack of a standardized method for distinguishing these two processes [10]. For example, the common technique of treating adipocytes in suspension with collagenase before measuring their sizes by flow cytometry [11,12] biases towards smaller cells due to the rupture of the more fragile large adipocytes and the parameters for adipocyte selection [10]. To further confound matters, crown-like structures, which are dead or dying adipocytes associated with increased adipocyte turnover [13,14], result in populations of smaller adipocytes that can easily be confused with *de novo* adipocytes associated with hyperplasia despite being the result of distinct metabolic processes. Therefore, the need for a robust method to evaluate adipocyte size within the context of the entire adipose tissue is critical.

A crucial factor to the consistency and reproducibility of adipocyte size measurements is a large sample size, or the number of cells accurately identified in each tissue. Since the advent of adipocyte size evaluation in the early 1970s [15,16], the field has improved in this metric by incorporating emerging techniques in image acquisition and analysis. A shift from manual to automated image analysis started in the early 2000s with the introduction of simplistic algorithms that identified adipocytes as separate units within one image, a process known as segmentation [11,17]. However, the sample size remained relatively low because the process of acquiring micrographs (digital cameras fitted to microscopes) was primarily manual and extremely time-consuming. The introduction of slide scanners in the early 2010s led to a sharp increase in sample size for the same amount of time investment, since more micrographs could be obtained in a semi-automated fashion across each tissue sample [18–20].

In the last 5–7 years since the most recent whole-slide analyses were published, new issues have come to light. Software for acquisition and quantification is now often proprietary, expensive, and complicated for users without computer science experience. Open-source alternatives validated by publications from the early 2000s have become incompatible with newer computer systems or are simply no longer available at their published hyperlinks, and no new plugins incorporating whole-slide technology have been published since 2014. Therefore, many investigators default to the inexpensive and previously accepted method of acquiring 1–5 micrographs per *in situ* slide and measuring areas with in-house or open-source plugins [21–24]. However, sample sizes remain low, time costs remain high, and there is a lack of consistency between research groups. Recent advances in affordable slide scanners, whole-slide image analysis, and open-source software could mitigate these complications, but a validation of these modern methods has yet to be published.

In this study, we compared three *in situ* methods to determine adipocyte count and size from histological tissue samples: the traditionally accepted analysis of a limited number of micrographs per slide, a whole-slide image analysis pipeline with open-source software, and whole-slide analysis with proprietary software (hereafter termed micrograph, QuPath, and Visiopharm, respectively). We developed an interactive plugin for adipocyte measurement with the novel software QuPath, which was introduced in 2016 as an open-source alternative to proprietary whole-slide image analysis software [25]. Following previously established validation procedures [17–19], we analysed tissue from mice fed control (Chow) or high-fat diets (HFD) to confirm that images could be segmented successfully into adipocytes and that adipocyte sizes increased with HFD as expected. Finally, we conducted a detailed inter-method comparison of sample sizes and adipocyte area measurements, clearly demonstrating the benefits of whole-slide over micrograph analysis.

## 2. Methods

### 2.1 Animal and sample preparation

All animal studies were approved by the university Institutional Animal Care and Use Committee (IACUC) prior to initiation and all methods were performed in accordance with relevant guidelines. Male C57Bl/6 N mice, aged 6 weeks, were obtained from Harlan Laboratories (Somerville, NJ) and were housed one per cage under 12:12 light:dark conditions at a temperature of 22°C in a humidity-controlled room. Mice were provided with standard laboratory chow and water *ad libitum* for a one-week acclimatization period before beginning experimental diets. After acclimatization, one group of mice remained on the chow diet while another was given *ad libitum* access to a high-fat Western diet (HFD, Test Diets, Cat. #5TJN). The HFD contained 40% energy from fat and was composed of 30% lard, 30% butterfat and 30% Crisco. After 12 weeks of dietary exposure, mice were euthanized by inhalation of CO_2_. Epididymal white adipose tissue (eWAT) was excised on ice and weighed. The mean weight and standard deviation of eWAT tissue from chow-fed and HFD-fed mice were 1.22 g ± 0.55 and 2.17 g ± 0.39, respectively. Portions of the eWAT were immediately fixed in 10% neutral buffered formalin for a minimum of 48 hours, processed overnight, paraffin-embedded, sectioned at a thickness of 5 µm, then stained with haematoxylin and eosin (H&E).

### 2.2 Micrograph quantification

To mimic conventional, well-accepted methods of measuring adipocyte number and size, we used an Olympus BX43 microscope with an Olympus DP27 camera to obtain one representative micrograph of each sample at 10x magnification (cellSense imaging software; Olympus Corporation, Japan). We obtained manual counts of the number of adipocytes in each image identifiable by a trained observer. We also loaded the images into ImageJ for automated counting using the MRI Adipocyte Tools plugin (See Supplementary Methods for step-by-step details). We first defined our desired adipocyte size range (500–20,000 µm^2^), then removed background by setting the number of dilates, or connections among defined adipocytes, to 10. We segmented adipocytes within the size specifications using ‘Percentile’ thresholding, followed by the ‘Simple Segmentation’ command to count the number of cells within an image. A board-certified pathologist reviewed all of the slides and associated analyses to confirm appropriate identification of adipocytes. We then used the ROI Manager to determine the area of each detected adipocyte. This generated a Results window from which we recorded the area of each adipocyte and the mean adipocyte area, minimum adipocyte size, and maximum adipocyte size within each image.

### 2.3 QuPath quantification

We scanned the slides using an Aperio ScanScope at 40x magnification and stored them on a Leica server organized by Aperio eSlide Manager. Using Aperio ImageScope software, we extracted a region including all relevant tissue to a TIF file with LZW compression. We opened each file in QuPath (version 0.1.2) and used the Simple Tissue Detection feature to exclude extraneous whitespace (see Supplementary Methods for specific parameters). After sending to ImageJ using QuPath’s built-in extension, we ran our Adipocyte QuPath plugin (developed in-house, available in linked GitHub repository; github.com/asm0028/Adipocyte_QuPath) to identify cells with areas from 500 to 20,000µm^2^ and circularity from 0.3 to 1.0. This allowed the algorithm to exclude cells that were crushed or otherwise distorted. A board-certified pathologist reviewed all of the slides and associated analyses to confirm appropriate identification of adipocytes. The plugin works by allowing the user to input desired area and circularity ranges, running the ‘Find Edges’ command 3 times, turning the image binary, then using the ‘Analyze Particles’ command with the user-defined restrictions. We then recorded the Count and Average Size results from the Summary window and the area of each adipocyte from the Results window.

### 2.4 Visiopharm quantification

We imported the whole slide files (same as described above for the QuPath quantification) into Visiopharm software version 2017.2. We used the APP Author function to design an algorithm which first used simple thresholding to classify pixels with intensities from 0 to 230 as Membrane, and those from 231 to 255 as Fat. It then defined adipocytes by using a series of post-processing steps to fill holes in the membrane borders and eliminate noisy pixels (See Supplementary Methods for details). Finally, the algorithm eliminated adipocytes from analysis if they were outside the following parameters: Area 500–20,000 µm^2^, Perimeter 0–900 µm (perimeter used as a proxy for ImageJ’s circularity function that is deprecated in this version of Visiopharm). Similar to the QuPath quantification method, these steps resulted in the exclusion of crushed regions and other artefacts, as verified by a board-certified pathologist.

### 2.5 Data analysis

All statistical analyses and graphs were generated with GraphPad Prism version 7.0d for Mac OSX (GraphPad Software, La Jolla California USA, www.graphpad.com).

Cell Count and Areas: For each slide, we reported the number of successfully segmented cells as an adipocyte-specific sample size (n), and the areas of each cell were then averaged to give a mean adipocyte area (μm^2^). These values (cells counted and mean area) were then averaged over each diet group, Chow or HFD. We used the nonparametric Kruskal–Wallis test, followed by Dunn’s multiple comparisons tests, to compare adipocyte areas between methods and diets. For cell count analysis, we used Students t-tests to determine significance between the two micrograph count methods (manual vs ImageJ) independently from the two whole-slide count methods (QuPath vs Visiopharm), and to compare the Chow to HFD groups within each method.

#### 2.5.1. Inter-method comparisons

To compare all three methods to each other on an adipocyte population level, we generated histograms sorted by area with a bin size of 500 µm. To create relative frequency plots, we normalized the number of counted cells per bin to the total number of cells counted per image. Pairwise comparisons of the methods were performed through Bland-Altman and regression plots. Each point on a Bland-Altman plot represents one sample that has undergone both methods of analysis. The x-axis plots the average and the y-axis plots the difference between the values given by each method.

## 3. Results

### 3.1 Whole-slide image analysis successfully segments more adipocytes than micrograph analysis, particularly in tissue from HFD-fed mice.

**Figure 1.**
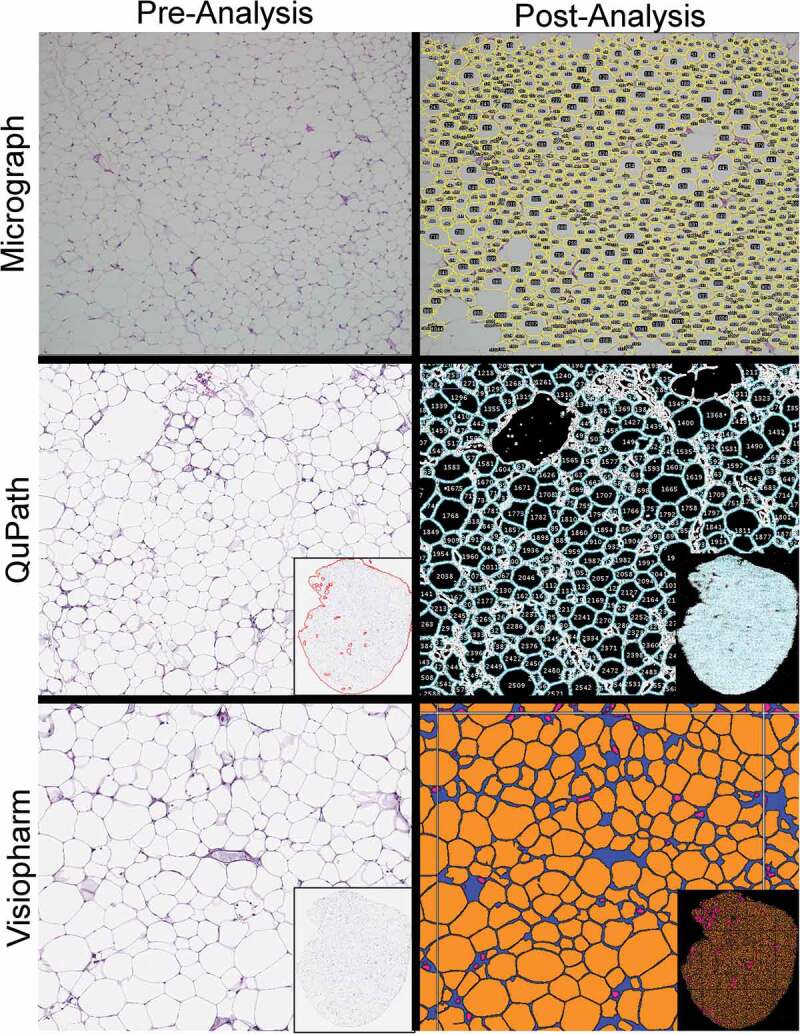
Screenshots of adipocyte tissue images pre- and post-analysis

Based on the established inclusion criteria for adipocyte identification, all three automated methods successfully segmented individual adipocytes from the given image and calculated their areas (Figure 1). Approximately 2.1%, 31%, and 49% of the total tissue area that was available on the slide for assessment was both evaluated and correctly identified as adipocytes using the micrograph, QuPath, and Visiopharm methods, respectively. Additionally, the whole-slide image methods successfully excluded material that was outside the set parameters for size and circularity. Visual review of these areas confirmed the excluded tissue as vessels, inflammatory cells, or distorted or crushed adipocytes.

Each method successfully segmented adipocytes and calculated their areas, which are shown in yellow outlines (micrograph), blue outlines (QuPath), or as solid orange (Visiopharm). Magnification: Micrograph 4x, QuPath 5x, Visiopharm 5x. Insets: whole-slide subgross images when applicable.

As expected, both whole-slide image analysis methods counted significantly more adipocytes than the micrograph analysis (Table 1), because more tissue area was evaluated. While the difference was apparent with the Chow group (QuPath and Visiopharm counted ~10x and ~18x more than the micrograph method, respectively), it became striking with the HFD group, where QuPath and Visiopharm counted ~30x and ~40x more cells, respectively. The cell count was more in agreement between the two whole-slide methods, with Visiopharm counting ~1.5x more cells than QuPath. Differences in cell count were also noted within each method, with a ~ 50% decrease for the micrograph method between Chow and HFD in contrast to a ~ 1.5x increase for both whole-slide methods. The manual count in micrographs was not statistically different from the ImageJ count (p = 0.58), providing another metric to validate the ImageJ algorithm.

**Table 1.** Number and average area of individual adipocytes by method

	Cells counted (n)		Average area (µm^2^)	
	*Chow*	*High Fat Diet*	*Chow*	*High Fat Diet*
Micrograph (manual)	208 ± 56	168 ± 35	N/A	N/A
Micrograph (Image J)	259 ± 129	155 ± 51	1280 ± 551	4987 ± 1450*^a^*
QuPath	2502 ± 1272	4511 ± 2863	2223 ± 640*^b^*	3320 ± 309*^a^*
Visiopharm	4633 ± 2047 *^c^*	6370 ± 3496	2367 ± 462*^b,c^*	3234 ± 373*^a,b,c^*

*^a^p*-value <0.0001 compared to Chow average area of same method.

*^b^p*-value <0.05 compared to Micrograph analysis of same diet.

*^c^p*-value <0.05 compared to QuPath analysis of same diet.

Values are mean ± SD

In addition, we observed a significant increase in average adipocyte area for the HFD group compared to the Chow group using all three methods. Micrograph analysis using a common approach estimated the mean cell area to be significantly smaller (0.56x) than both whole-slide methods for the Chow group, but much larger (1.5x) for the HFD group. However, the high standard deviation for the micrograph method, especially for HFD-fed mice, lowers the likelihood that these mean areas are accurate values. The whole-slide methods reported similar values to each other; though they are statistically different, this is likely due to the relatively high power of the comparison (n ~ 17,500 for QuPath and ~32,000 for Visiopharm).

### 3.2 Micrograph analysis of cell area yields a substantially higher variance than both whole-slide analysis methods.

**Figure 2.**
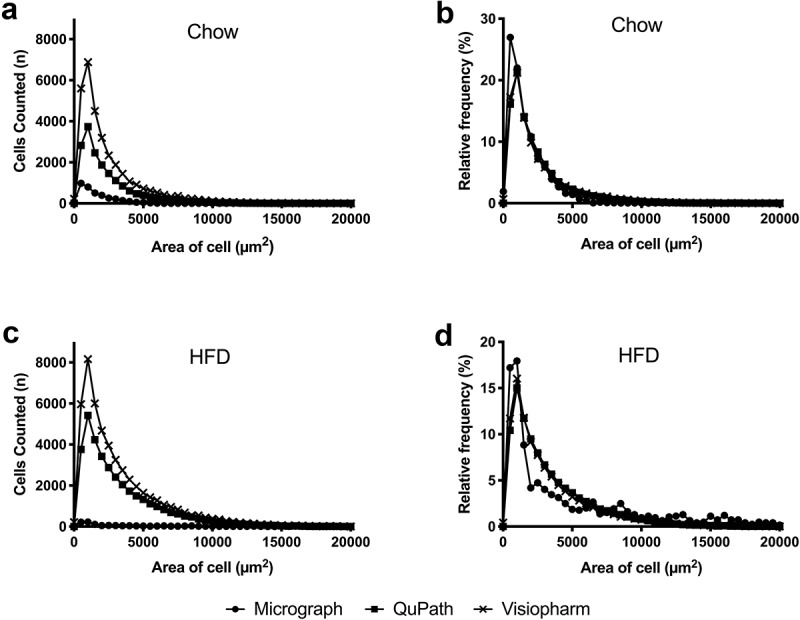
Population-level representations of adipocyte size

To evaluate the differences in adipocyte size on a population level, we generated histograms and distribution curves of counted cells sorted by area (bin = 500 µm^2^) for each diet and method (Figure 2). The histograms represent the absolute number of cells of a given size counted for each bin (Figure 2(a,c)), while the distribution curves represent a relative frequency in which cells of a given size per bin were normalized to the total number of cells counted per slide (Figure 2(b,d)). Both HFD curves (Figure 2(c,d)) display a higher proportion of adipocytes in size bins >5,000 µm^2^ compared to Chow (Figure 2(a,b)), as expected for animals on this diet, and corresponding to data shown in Table 1. The QuPath and Visiopharm distribution curves have nearly identical smooth shapes, implying that there are enough values in each bin to depict the overall trend accurately (Figure 2(b,d)). In contrast, the micrograph distribution curves display multiple deviations from an ideal smooth shape, with the HFD data especially generating an irregular overall shape. This indicates that micrograph analysis results in a high variance, likely due to its small sample size (Table 1), and increases the chance that areas measured are not representative of the entire adipocyte population within each tissue. Therefore, studies that investigate the effects of HFD and/or obesity through the micrograph method may be prone to more statistical errors and false conclusions unless their sample sizes increase substantially.

Histograms with absolute numbers of adipocytes of a given size were sorted by area with bin size of 500 µm^2^ (a, c). Relative frequency plots: the number of counted cells of a given size per bin (size = 500 µm^2^) was normalized to the total number of cells counted per slide to generate a percentage (b, d). Slides from Chow-fed mice (n = 7) are shown in A and B, while slides from HFD-fed mice (n = 8) are shown in C and D.

### 3.3 Whole-slide analysis methods are more consistent than micrograph analysis at evaluating mean adipocyte size, particularly as adipocyte size increases.

**Figure 3.**
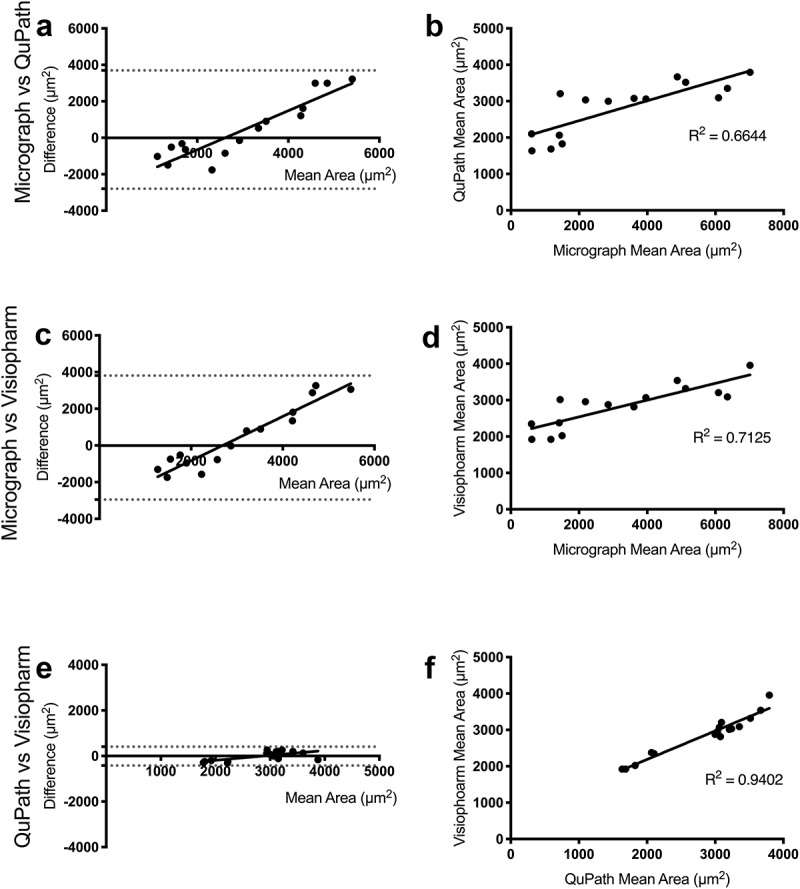
Pairwise representations between image analysis methods

The Bland-Altman plots demonstrate that the agreement between the micrograph method and each of the whole-slide analyses is much lower than the agreement between the two whole-slide methods. This is indicated by ~8x larger 95% limits of agreement (6,492 and 6,760 for micrograph versus QuPath and Visiopharm, respectively, and 826 for QuPath versus Visiopharm) (Figure 3(a, c, e)). Further evidence for a lack of agreement is shown in the linear regression plots (Figure 3(b, d, f)), in which the R^2^ values for the plots of ImageJ versus QuPath and Visiopharm are lower (0.6644 and 0.7125, respectively) compared to the R^2^ of QuPath versus Visiopharm (0.9402). This disagreement increases with adipocyte size (Figure 3(a, c)), which corresponds with the high variability in large adipocyte measurements observed with micrograph analysis (Figure 2(d)). In contrast, the small 95% limit of agreement and large R^2^ value between QuPath and Visiopharm indicate minimal differences between the methods regardless of cell size.

Bland-Altman plots of micrograph and QuPath (a), micrograph and Visiopharm (c) and QuPath and Visiopharm (e) emphasize the greater difference in method results between micrograph and whole-slide image analysis. Regression plots indicate a weak correlation between QuPath and micrograph methods (b), a weak but greater correlation between Visiopharm and micrograph methods (d) and a strong correlation between Visiopharm and QuPath, the two whole-slide image analysis methods (f).

## 4. Discussion

This study is the first to validate and compare open-source and proprietary whole-slide image analysis pipelines for measuring adipocyte size and demonstrate their clear advantages over the more traditional micrograph method. Micrograph analysis is limited by its restriction to finite portions of each slide, which significantly decreases the adipocyte sample size and therefore the likelihood that cell sizes of the population are accurately estimated. Because the micrograph cell count was consistent between manual and ImageJ methods, this small sample size is attributed to the limited area available for analysis in micrographs, as opposed to errors in the ImageJ algorithm. This problem was exacerbated with the increased proportion of hypertrophied adipocytes in the HFD-fed mice, since fewer large cells can physically fit within the limited area of each image. Whole-slide analysis removed this limitation entirely, and in fact the opposite trend was observed (more cells counted in the HFD group than the Chow group). This difference is consistent with the increased overall adipose tissue mass that is associated with HFD feeding. In turn, the larger tissue samples improved ease of handling and generated relatively less crush artefact, which could be accounted for and eliminated by the whole-slide algorithms.

The small adipocyte sample size of the micrograph method had several downstream consequences that resulted in a decreased likelihood of accurate area measurements. The irregular shape of the distribution curves indicated a high variance within the micrograph method, and the Bland-Altman and correlation plots demonstrated its lower agreement with both whole-slide analyses. Another contributor to this variability could be sample selection bias, since most users have a natural desire to maximize the number of intact cells in each image. For example, if there is less crush artefact in the centre of the tissue versus on the periphery, the user may choose to focus on this region and exclude other more variable regions. There is also a risk for double-counting the same adipocytes if the fields of view overlap between micrographs. These factors all contribute to a low probability that similar measurements could be achieved from micrographs acquired elsewhere in the same tissue sample, which substantially affects reproducibility and consistency between research groups. The fact that this decreased accuracy is exacerbated with HFD-fed mice is particularly concerning because investigations into metabolic derangements such as obesity and type-2 diabetes mellitus often examine adipocyte hypertrophy, hyperplasia and adipose tissue remodelling. Therefore, though the micrograph method in our study did successfully detect the expected increase in adipocyte size with HFD-fed mice, the high variance raises significant concerns regarding its ability to accurately detect more subtle differences. These changes are crucial to evaluating therapeutic effects within tissues from HFD-fed or obese subjects.

The impressive agreement between the QuPath and Visiopharm methods, which use different types of image analysis algorithms, validates both methods and allows for a detailed comparison of their implementations. Both whole-slide methods generated large enough sample sizes that their variances were low and therefore their probability of accurate size calculations was high. The Visiopharm algorithm was based on simple thresholding and extensive post-processing, a procedure that was customized to our specific tissue characteristics (and therefore resulted in the highest absolute cell count) but would likely need major revisions to implement on tissue processed elsewhere. We found that the creation of more complex algorithms was difficult in this software since it was designed for the automation of common pathology tasks, and customization therefore required disassembling existing pipelines. In addition, the proprietary nature of Visiopharm makes it difficult to access algorithms previously developed by investigators asking similar questions, and the company support, while useful, does not encourage a robust online community of users.

In contrast, the QuPath algorithm, which worked by downsampling the extremely large whole-slide images before relying on the ImageJ command ‘Find Edges’, resulted in a lower cell count than Visiopharm but may translate more easily between research groups. The more minimal user interface of QuPath and ImageJ is intended to provide building blocks for customization rather than ‘out-of-the-box’ utility, which is sometimes intimidating to new users. However, the open-source nature of QuPath allows direct sharing of plugins and the creation of a large online community where researchers are likely to find guidance from those who have worked through similar issues. While we provide the plugin used in this paper (see GitHub repository; https://github.com/asm0028/Adipocyte_QuPath), we recognize that tissue processing, experimental parameters, and target species frequently change among research groups. Therefore, we encourage investigators to use this plugin as a starting point from which they can make specific modifications and explore the possibilities of open-source software, rather than considering it a static end product. In particular, we expect its translation to human adipose tissue to be both feasible and important for the field.

One more crucial factor that investigators consider when selecting appropriate methods for their research questions is cost: both financial and time/labour. The micrograph method requires the least amount of upfront financial investment, since a microscope fitted with a camera is the only expensive equipment and many research groups already access it. While both whole-slide methods require a slide scanner and storage system for large image files, this equipment is becoming more standardized as the price continues to come down. The Visiopharm method incurs additional financial costs for the proprietary software and a computer with the significant processing power required to load and display the sizable image files. In contrast, open-source software is free to download and typically only requires the processing power provided by most modern computers. As for time and labour costs, all methods required a similar amount of time to optimize their respective algorithms and process each image. However, the only way to increase the deficient sample size of the micrograph method is to acquire and analyse more images, which causes a direct and substantial increase in time and labour investment. That investment and technical issues are then compounded for every additional project involving adipose tissue image analysis, in contrast to the whole-slide pipelines that are more easily adapted to future studies.

In conclusion, the small sample size and decreased accuracy of the micrograph method may lead to false conclusions when evaluating samples from obese subjects or animals fed a HFD. This leads us to recommend more modern whole-slide image analysis techniques, especially when evaluating the size of hypertrophied adipocytes. To address this problem and provide support, we created and shared a novel open source image analysis plugin for this purpose and encourage investigators to adapt it to their own adipocyte measurement needs. Future directions of adipose tissue whole-slide image analysis should include enhancing algorithm standardization between research groups and quantifying other important structures smaller than the 500 µm^2^ minimum in this study, such as crown-like structures and beige, brite, and brown adipocyte subtypes. These techniques may naturally become more adaptable as new technologies such as artificial intelligence and deep learning for image analysis become more readily available. As new methods are developed to evaluate large numbers of adipocytes *in situ*, new insights will be gained into the complex ways in which adipose tissue responds to common metabolic derangements.

## Supplementary Material

Supplemental Material
