# Supplementary material for: Whole-slide image analysis outperforms micrograph acquisition for adipocyte size quantification
Source: Adipocyte. 2020 Sep 20;9(1):567–75. doi: 10.1080/21623945.2020.1823139 (PMC7714435; doi:10.1080/21623945.2020.1823139)
Supplement: Supplemental Material [file KADI_A_1823139_SM8159.pdf]

Supplementary Methods

Micrograph Quantification

- The ImageJ plugin can be found at: https://github.com/MontpellierRessourcesImagerie/imagej_macros_and_scripts/wiki/Adipocytes-Tools
- Step-by-step:
  1. Download MRI Adipocyte Tools to ImageJ toolsets folder.
  2. Right-click on the "p" button to open the Preprocessing Adipocytes Segmentation Options dialog box. Define minimum size, maximum size, number of dilates and thresholding method.
     a. Min. size = 500 um
     b. Max. size = 20000 um
     c. Nr. Of dilates = 10
     d. Thresholding method = Percentile.
  3. Click "OK" to confirm Preprocessing settings.
  4. Left-click "p" to run Preprocessing.
  5. Use the Simple Segmentation Method:
     a. Click on "s" to run.
     b. ROI Manager dialog box will appear when analysis is done.
     c. Select all ROIs.
     d. Click on "Measure".
     e. Results dialog box will appear with Area, Mean, Min and Max data for each measured cell.
     f. Select all measurements and copy to Excel.

QuPath Quantification

- *Parameters for Simple Tissue Detection*: Threshold: from 233-243 to best fit tissue, Requested Pixel Size: 20µmm, Minimum area – 10,000 µm^2, Max fill area – 20,000 µm^2, Dark background – no, Smooth image – yes, Cleanup with median filter – yes, Expand boundaries – no, Smooth coordinates – yes, Exclude on boundary – no, Single annotation – yes
- *Parameters for Adipocyte QuPath Plugin*: Area range – 500-20,000 µm^2, Circularity range – 0.30-1.00, Inversion – no unless adipocytes were not counted with first attempt, then yes for second attempt

Visiopharm Quantification

1. Setup
   a. Magnification 10x
   b. Fraction 99%
2. Preprocess
   a. RGB-G checked
   b. RGB-R and RGB-B unchecked
3. Classify
   a. Method: Threshold
   b. Label: Membrane
      i. Feature: RGB-G, range (0-230)
      ii. [else: Fat label, but can't see where this is inputted?]
4. Post process
   a. Change by Shape

        i. Property: Area
        ii. Label: Membrane
        iii. Replace with: Fat
        iv. Min: blank
        v. Max: 100µm^2
    b. Change by Shape
        i. Property: Area
        ii. Label: Fat
        iii. Replace with: Membrane
        iv. Min: blank
        v. Max: 50µm^2
    c. Change Surrounded
        i. Label: Fat
        ii. Surrounded by: Membrane
        iii. Coverage 0.8
        iv. Replace with: Adipocyte
    Unchecked: Include image border
    d. Separate Objects
        i. Object label: Adipocyte
        ii. Separator label: Membrane
        iii. Background labels: All except object
        iv. Object diameter: 1000 µm
        v. Separate object surroundings: unchecked
        vi. Assume that objects are elliptic: checked
        vii. Object heatmap: Don't use
    e. Close
        i. Object label: Membrane
        ii. With: Membrane
        iii. Pixels: 10
        iv. Exclude labels: none selected
    f. Change by Shape
        i. Property: Area
        ii. Label: Adipocyte
        iii. Replace with: Second Adipocyte
        iv. Min: 500µm^2
        v. Max: 200000µm^2
    g. Change by Shape
        i. Property: Perimeter
        ii. Label: Second Adipocyte
        iii. Replace with: FINAL Adipocyte
        iv. Min: 0µm
        v. Max: 900µm
    h. Separate Objects
        i. Object label: FINAL Adipocyte
        ii. Separator label: Membrane
        iii. Background labels: All except object
        iv. Object diameter: 1000µm
        v. Separate object surroundings: unchecked
        vi. Assume that objects are elliptic: checked
        vii. Object heatmap: Don't use
5. Calculations step

a. Parameter: Count of Label: FINAL Adipocyte
b. Parameter: Area of each Label: FINAL Adipocyte
